# Molecular Regulators of Muscle Mass and Mitochondrial Remodeling Are Not Influenced by Testosterone Administration in Young Women

**DOI:** 10.3389/fendo.2022.874748

**Published:** 2022-04-14

**Authors:** Oscar Horwath, Marcus Moberg, Angelica Lindén Hirschberg, Björn Ekblom, William Apró

**Affiliations:** ^1^ Department of Physiology, Nutrition and Biomechanics, Åstrand Laboratory, Swedish School of Sport and Health Sciences, Stockholm, Sweden; ^2^ Department of Physiology and Pharmacology, Karolinska Institutet, Stockholm, Sweden; ^3^ Department of Women´s and Children´s Health, Division of Neonatology, Obstetrics and Gynaecology, Karolinska Institutet, Stockholm, Sweden; ^4^ Department of Gynaecology and Reproductive Medicine, Karolinska University Hospital, Stockholm, Sweden; ^5^ Department of Clinical Science, Intervention and Technology, Karolinska Institutet, Stockholm, Sweden

**Keywords:** androgen receptor, mTORC1-signaling, ubiquitin-proteasome system, fission, fusion

## Abstract

Testosterone (T) administration has previously been shown to improve muscle size and oxidative capacity. However, the molecular mechanisms underlying these adaptations in human skeletal muscle remain to be determined. Here, we examined the effect of moderate-dose T administration on molecular regulators of muscle protein turnover and mitochondrial remodeling in muscle samples collected from young women. Forty-eight healthy, physically active, young women (28 ± 4 years) were assigned in a random double-blind fashion to receive either T (10 mg/day) or placebo for 10-weeks. Muscle biopsies collected before and after the intervention period were divided into sub-cellular fractions and total protein levels of molecular regulators of muscle protein turnover and mitochondrial remodeling were analyzed using Western blotting. T administration had no effect on androgen receptor or 5α-reductase levels, nor on proteins involved in the mTORC1-signaling pathway (mTOR, S6K1, eEF2 and RPS6). Neither did it affect the abundance of proteins associated with proteasomal protein degradation (MAFbx, MuRF-1 and UBR5) and autophagy-lysosomal degradation (AMPK, ULK1 and p62). T administration also had no effect on proteins in the mitochondria enriched fraction regulating mitophagy (Beclin, BNIP3, LC3B-I, LC3B-II and LC3B-II/I ratio) and morphology (Mitofilin), and it did not alter the expression of mitochondrial fission- (FIS1 and DRP1) or fusion factors (OPA1 and MFN2). In summary, these data indicate that improvements in muscle size and oxidative capacity in young women in response to moderate-dose T administration cannot be explained by alterations in total expression of molecular factors known to regulate muscle protein turnover or mitochondrial remodeling.

## Introduction

Testosterone (T) is a well-known anabolic agent which promotes dose-dependent increases of lean mass and muscle hypertrophy in young and old men (1–3). Similarly, we and others have previously shown that administration of exogenous T promotes muscle anabolism and lean mass accretion in pre- and post-menopausal women (4–6). However, despite compelling evidence of its anabolic effect, the molecular events underlying muscle hypertrophy following T exposure in humans are not well understood and data from female-only cohorts are lacking.

Given its role as a critical regulator of muscle protein synthesis (7, 8), the mechanistic target of rapamycin complex 1 (mTORC1) pathway is considered a candidate mediator of T-induced muscle growth (9). Indeed, provision of T in cultured muscle cells stimulates hypertrophy *via* mTORC1-dependent signaling (10, 11). This is further supported by rodent data showing reduced signaling activity downstream of mTORC1 after androgen deprivation, while restoring androgen levels by nandrolone injections (6 mg/kg bw) reversed this effect (12). Studies seeking to depict the role of mTORC1-signaling in human muscle following T provision are scarce and have provided mixed results. Howard et al., 2020 showed that weekly injections of T enanthate (200 mg) during 28-days of severe energy deficit did not alter mTORC1-signaling following exercise and protein intake in young men (13). On the other hand, Gharahdaghi and colleagues showed that biweekly T injections (Sustanon, 250 mg) in combination with resistance training potentiated exercise-induced mTORC1-signaling in old men (14). More research in human muscle is although required to verify whether a similar mechanism of action is present in other cohorts.

Changes in muscle mass are dictated by the finely tuned balance between synthesis and degradation of muscle protein (15). Suppression of the latter is assumed to be central for driving lean mass accretion in humans following T exposure (16–18). Mechanistically, protein degradation occurs primarily through the autophagy-lysosomal and the ubiquitin-proteasomal system (UPS) (19). The UPS involves two well characterized muscle specific ubiquitin ligases; Muscle Atrophy F-box (MAFbx) and Muscle RING-finger 1 (MuRF-1) (20, 21). The existing knowledge of how T provision influences markers of UPS-mediated protein degradation is although limited, and inconclusive results have been reported. In the study by Gharahdaghi and colleagues, MAFbx or MuRF-1 protein levels remained unchanged (14), whilst a reduction of these markers were observed in hypogonadal men following T replacement therapy (TRT) (50-100 mg daily) (22). Moreover, whether the autophagy-lysosomal pathway is influenced by androgen levels in human muscle remains to be explored. The increased re-utilization of intracellular amino acids observed after T provision might however represent a putative mechanism for how autophagy could prevent a negative protein balance in the fasted state and thereby contribute to muscle growth (17, 23). In rodents, muscle atrophy induced by castration is associated with elevated levels of microtubule-associated protein 1A/B-light chain 3B (LC3B)-II and polyubiquitin-binding protein 62 (p62), whilst the re-introduction of androgens to the circulation restored their expression levels (24, 25). As such, there is evidence that the autophagy pathway is responsive to changes in systemic androgen concentrations, however, these findings may not be readily translatable to human physiology under *in vivo* conditions where androgen concentrations are less altered in response to exogenous supplementation.

We have previously demonstrated that administration of T increased oxidative capacity in human muscle by improving respiration in isolated mitochondria, a measure of mitochondrial quality (26). However, since this was not paralleled by concomitant increases in mitochondrial protein abundance, i.e., citrate synthase and respiratory chain complex I-V, other factors, such as alterations in mitochondrial remodeling, may underlie these changes. Intriguingly, systemic androgen levels are shown to impact the abundance of proteins involved in mitochondrial quality control in mouse muscle (27, 28), and exposure to high doses of T (50 mg/kg bw) upregulated several key proteins associated with mitophagy as well as fission and fusion (29). While prior work has focused on mitochondrial biogenesis in human muscle following T provision (14, 30), molecular regulators of the remodeling machinery have previously not been addressed in this context.

The molecular mechanisms responsible for adaptations in human skeletal muscle following T administration are not fully understood and the translatability from cell and rodent studies is often limited. Furthermore, understanding how mitochondrial turnover is affected by alternating T concentrations may have clinical implications for mitigating declines in muscle mass and function during aging. Therefore, in the present study we sought to investigate if the previously observed increases in muscle size and oxidative capacity following 10 weeks of T administration in young women are mediated by alterations in protein levels of molecular markers regulating muscle protein turnover and mitochondrial remodeling.

## Material and Methods

### Ethical Approval

This study is part of a larger project investigating the impact of T administration on physical performance and muscle mass in young physically active women, registered at ClinicalTrials.gov (NCT03210558). This study was approved by the local ethics committee in Stockholm (2016/1485-32, amendment 2017/779-32), and was conducted in line with the principles outlined in the Declaration of Helsinki. All participants received detailed information about the design of the study and associated risks before providing their written consent.

### Study Design

The design of the present study has been described in detail in our previous publications (4, 26, 31). Briefly, this was a randomized, double-blinded, placebo-controlled trial. Following initial screening procedures, participants were randomly allocated to 10-weeks of either T or placebo treatment. T was administered by manually applying a gel (10 mg, Andro-Feme 1) to the outer thigh every evening. This dose was chosen to increase serum levels of T significantly above the physiological level without inducing harmful side effects. Participants were instructed to keep their habitual activity levels throughout the study period, and they were also informed not to make any changes to their diet.

### Participants

Forty-eight young (28 ± 4 years), physically active, women participated in the present study. Physical characteristics and serum levels of T have been reported previously (4, 26, 31).

### Muscle Biopsy Sampling

Muscle samples were collected ~ 60 min after the participants finished a physical testing session (26, 31). Muscle biopsies were obtained from the middle portion of *m. vastus lateralis* after administration of local anesthesia using the Weil-Blakesley conchotome technique (32). Tissue samples were immediately blotted free of blood and rapidly frozen in liquid nitrogen until further processing.

### Muscle Tissue Processing

Muscle samples were freeze-dried overnight and dissected free of blood, fat, and connective tissue under a stereo microscope (VisiScope, VWR). Fibre bundles were then mixed carefully and stored in -80°C before further processing.

### Immunoblotting Sample Preparation

To study protein levels in whole muscle homogenates, ~ 2 mg of freeze-dried muscle was homogenized in ice-cold buffer (100 µl · mg^-1^ dry weight) containing 250 mM Sucrose, 20 mM HEPES (pH 7.4), 10 mM KCl, 1.5 mM MgCl_2,_ 1 mM EDTA, 1 mM EGTA, 10 mM β-glycerophosphate, 1% phosphatase inhibitor cocktail (Sigma P-2850) and 1% (v/v) Halt Protease Inhibitor Cocktail (Thermo Scientific, Rockford, USA) using a Bullet-Blender™ (NextAdvance, New York, USA) and 0.5 mm Zirconium Oxide Beads (NextAdvance, New York, USA). The supernatant was collected after lysates had been rotating for 60 min at 4°C and centrifuged at 10,000 g for 15 min. Protein concentration was determined from a small aliquot of the supernatant using Pierce™ 660 nm protein assay (Thermo Scientific, Rockford, USA). The samples were diluted in 4x Laemmli sample buffer (Bio-Rad Laboratories, Richmond, USA) and homogenization buffer, rendering a protein concentration of 0.75 µg µl^-1^. The samples were heated to 95°C for 5 min before being stored in -30°C.

To study protein levels specifically in the mitochondrial compartment, we fractionated muscle according to a previously described protocol with minor adjustments (33). In brief, ~ 6 mg of freeze-dried muscle was homogenized in ice-cold buffer (100 µl · mg^-1^ dry weight) containing 250 mM Sucrose, 20 mM HEPES (pH 7.4), 10 mM KCl, 1.5 mM MgCl_2,_ 1 mM EDTA, 1 mM EGTA, 10 mM β-glycerophosphate, 1% phosphatase inhibitor cocktail (Sigma P-2850) and 1% (v/v) Halt Protease Inhibitor Cocktail (Thermo Scientific, Rockford, USA), using a 2 ml glass Dounce tissue grinder set (Sigma, D8938), applying 10 and 30 strokes with pestle A and B, respectively. The tissue lysate was then transferred into new tubes, rotated for 60 min at 4°C and centrifuged at 1000 g for 10 min to allow for the formation of a myofibrillar pellet. The supernatant was then carefully transferred into new tubes and centrifuged at 16,000 g for 20 min, resulting in a cytosolic protein fraction. The remaining pellet was washed twice by carefully resuspending the pellet in homogenization buffer. Following the final centrifugation, the pellet was dissolved in 60 µl of homogenization buffer containing 1% TritonX-100, resulting in a membrane fraction (hereafter referred to as the mitochondrial fraction). This fraction was then combined with 20 µl of 4x Laemmli sample buffer (Bio-Rad Laboratories, Richmond, USA) and 400 mM dithiothreitol, heated to 37°C for 30 min, before being stored in -30°C. Protein concentration was determined using Ionic Detergent Compatibility Reagent (Thermo Scientific, Rockford, USA) and Pierce™ 660 nm protein assay (Thermo Scientific, Rockford, USA). To determine the purity of the mitochondrial fraction, 5 ug of protein from this fraction and the cytosolic fraction were loaded side-by-side on a gel and immunoblotted for Porin (VDAC1) and translocase of outer mitochondrial membrane 20 (TOM20), two proteins found in the outer mitochondrial membrane, and the eukaryotic elongation factor 2 (eEF2), a protein dispersed throughout the cytoplasm. As demonstrated in **Figure 3G**, eEF2 was only present in the cytosolic fraction whereas Porin and TOM20 were only detected in the mitochondrial fraction.

### SDS-PAGE and Immunoblotting

From each sample, 15 µg and 3 µg of protein from whole muscle homogenate and the mitochondrial fraction was loaded on 26-well Criterion TGX gradient gels (4-20% acrylamide; Bio-Rad Laboratories), respectively. Electrophoresis was performed on ice at 300 V for ~ 30 min. The gels were then equilibrated for 30 min in transfer buffer (25 mM Tris base, 192 mM glycine, and 10% methanol) after which proteins were transferred to PVDF membranes (Bio-Rad Laboratories) at constant current (300 mA) for 180 min at 4°C. Membranes were then stained using MemCode™ Reversible Protein Stain Kit (Thermo Scientific, Rockford, USA) to confirm even transfer of proteins. After destaining, membranes were blocked for 1h in Tris-buffered saline (TBS; 20 mM Tris base, 137 mM NaCl, pH 7.6) containing 5% non-fat dry milk followed by incubation overnight (4°C) with primary antibodies diluted in TBS supplemented with 0.1% Tween-20 and 2.5% non-fat dry milk (TBS-TM). Next morning, membranes were washed after which secondary antibodies conjugated to horseradish peroxidase were applied for 1h. Membranes were then washed again in TBS-TM (2 x 1 min, 3 x 10 min) followed by 3 x 5 min with TBS. Lastly, membranes were incubated with Super Signal™ Femto Chemiluminescent Substrate (Thermo Scientific) for 5 min to allow for band detection in the molecular imager (ChemiDoc™MP, Bio-Rad Laboratories). Before the blocking step, membranes were cut into strips and later assembled to expose all samples to the same blotting conditions. Due to limited quantity the mitochondrial fraction samples, following visualization, membranes were stripped using Restore Western Blot Stripping Buffer (Thermo Scientific) for 30 min at 37°C, washed and re-probed with a new primary antibody. For whole muscle homogenates, protein levels of each sample were normalized to their total protein stain. For the mitochondrial fraction, protein levels were normalized to the corresponding protein level of Porin, which remained unchanged throughout the intervention (data not shown). Quantification of bands were performed using the Image Lab™ software (Bio-Rad Laboratories).

### Antibodies

For immunoblotting, primary antibodies against androgen receptor (#D6F11XP^®^), mTOR (#2983), S6K1 (#2708), eEF2 (#2332), AMPK (#2532), ULK1 (#6439), p62 (#8025), LC3B (#2775), Beclin (#3495) and BNIP3 (#44060), were purchased from Cell Signaling Technology (Beverly, USA). Primary antibodies against 5α-reductase 2 (#293232), MuRF-1 (#sc-398608), UBR5 (#sc-515494), TOM20 (sc-136211) and RPS6 (#sc-74459), were purchased from Santa Cruz Biotechnology (Heidelberg, Germany). Primary antibodies against MAFbx (#92281), Porin (#154856), OPA1 (#157457), DRP1 (#184247), FIS1 (#156865), MFN2 (#56889) and Mitofilin (#137057), were purchased from Abcam (Cambridge, UK). All antibodies were diluted 1:1000 except for 5α-reductase, MuRF-1, UBR5, S6, Beclin, LC3B, OPA1, MFN2 which were diluted 1:500, and eEF2 and Porin which were diluted 1:2000. Secondary anti-mouse (#7076, 1:10000) and secondary anti-rabbit (#7074, 1:10000) were purchased from Cell Signaling Technology.

### Statistical Analyses

Data are presented as means ± standard deviation (SD). Statistical analyses were performed using GraphPad Prism version 9.1.2 for Windows (San Diego, California, USA). Data were analyzed with a two-way ANOVA with factors for group (T *vs*. placebo) and time (pre *vs* post intervention). Bonferroni multiple comparison was applied in case of significant interaction to localize differences. The significance level for all statistical tests was two-tailed and set at P < 0.05.

## Results

### Androgen Receptor, 5-α Reductase and Anabolic Signaling

**Figure 1:**
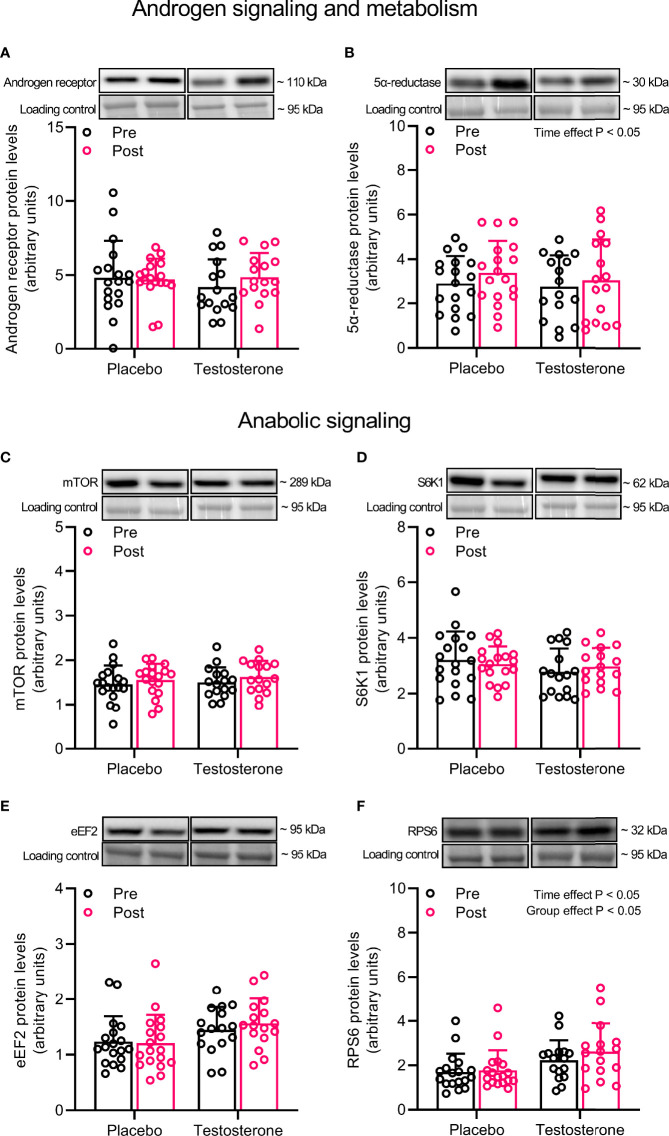
Protein levels of androgen receptor **(A)**, 5α-reductase **(B)**, mTOR **(C)**, S6K1 **(D)**, eEF2 **(E)** and RPS6 **(F)** before (black dots) and after the intervention (red dots). Representative blots for each individual protein target are shown above each graph. Loading control is represented by a band at ~ 95 kDa from the corresponding total protein stain (Memcode™). The values presented are means ± SD and individual data points from 18 and 16 individuals (DF 32) in the placebo group and T group, respectively. The ANOVA revealed a significant main effect of time with respect to changes in protein levels for 5α-reductase and a significant main effect of time and group for RPS6.

Administration of T had no effect on AR protein content but levels of 5-α reductase increased on average by 15% over time (main effect of time, P<0.05) (**Figures 1A, B**). In a similar fashion, administration of T did not influence the abundance of mTOR, S6K1 or eEF2, but levels of RPS6 increased on average by 11% over time and was overall higher in the group receiving T (main effect of time and group, P<0.05) (**Figures 1C–F**).

### Ubiquitin-Proteasomal and Lysosomal-Autophagy Pathway

**Figure 2:**
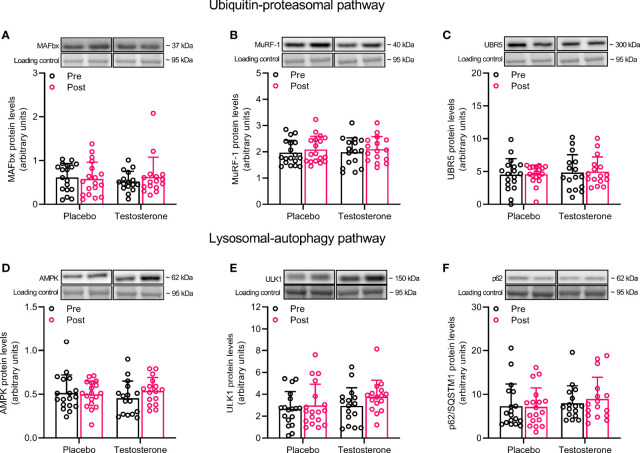
Protein levels of MAFbx **(A)**, MuRF-1 **(B)**, UBR5 **(C)**, AMPK **(D)**, ULK1 **(E)** and p62 **(F)** before (black dots) and after the intervention (red dots). Representative blots for each individual protein target are shown above each graph. Loading control is represented by a band at ~ 95 kDa from the corresponding total protein stain (Memcode™). The values presented are means ± SD and individual data points from 18 and 16 individuals (DF 32) in the placebo group and T group, respectively.

Administration of T did not alter protein levels of MAFbx, MuRF1 or UBR5 in the ubiquitin-proteasomal pathway or AMPK, ULK1 or p62 in the lysosomal-autophagy pathway (**Figures 2A–F**).

### Mitochondrial Remodeling

**Figure 3:**
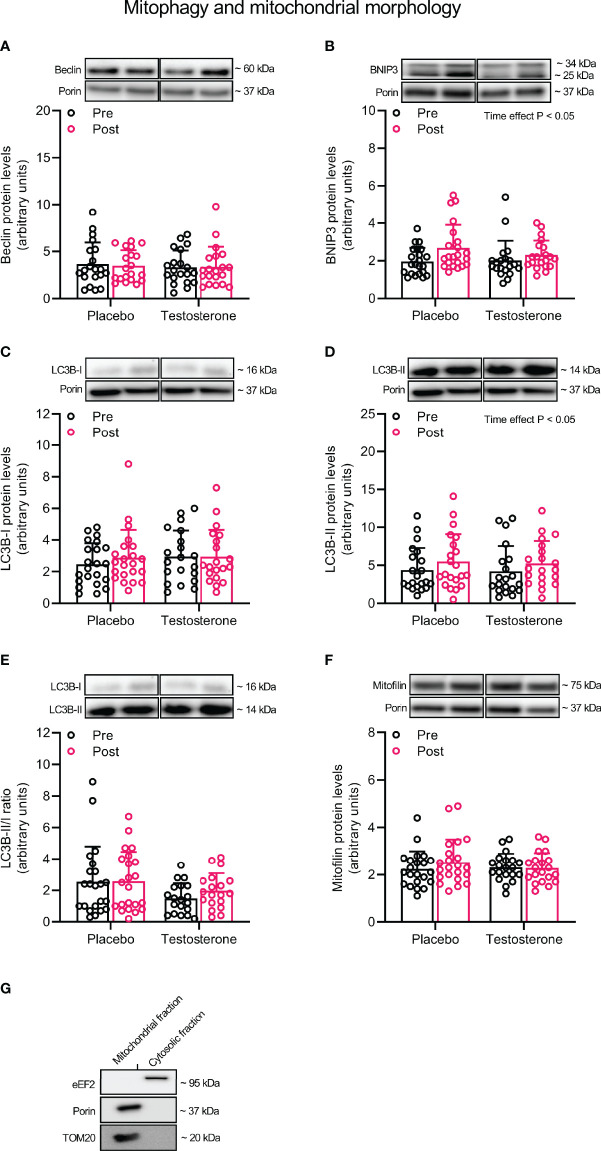
Protein levels of Beclin **(A)**, BNIP3 **(B)**, LC3B-I **(C)**, LC3B-II **(D)**, LC3B-II/I ratio **(E)** and Mitofilin **(F)** in the mitochondrial fraction before (black dots) and after the intervention (red dots), as well as the assessment of mitochondrial fraction purity **(G)**. Representative blots for each individual protein target and Porin are shown above each graph. The values presented are means ± SD and individual data points from 22 and 20 individuals (DF 40) in the placebo group and T group, respectively. The ANOVA revealed a significant main effect of time with respect to changes in protein levels for BNIP3 and LC3B-II. For illustrative purposes, two subjects from the placebo group displaying extreme values were removed from **(A)** (pre-post; 6.7 to 18.5 and 12.3 to 23.6, respectively) and one subject from the placebo group was removed from **(D)** (pre-post; 6.1 to 19.4), but these values were included in the statistical analysis.

**Figure 4:**
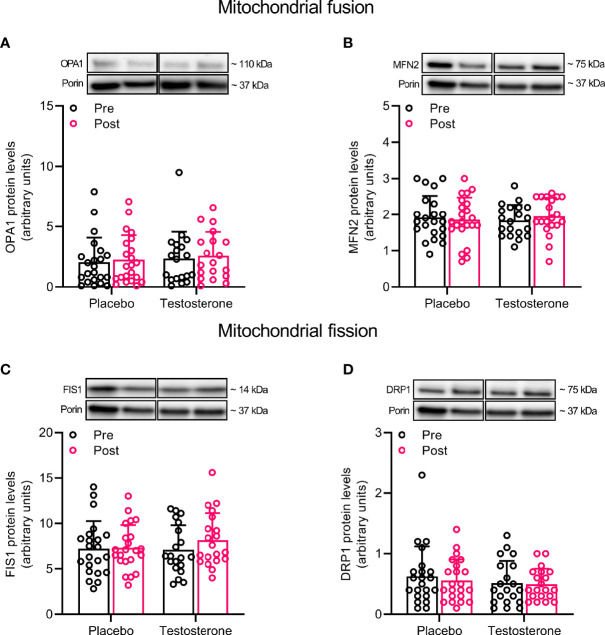
Protein levels of OPA1 **(A)**, MFN2 **(B)**, FIS1 **(C)**, and DRP1 **(D)** in the mitochondrial fraction before (black dots) and after the intervention (red dots). Representative blots for each individual protein target and Porin are shown above each graph. The values presented are means ± SD and individual data points from 22 and 20 individuals (DF 40) in the placebo group and T group, respectively. For illustrative purposes, one subject from the placebo group displaying extreme values was removed from **(A)** (pre-post; 6.1 to 19.4), but these values were included in the statistical analysis.

Administration of T had no effect on protein levels of Beclin and Mitofilin (**Figures 3A, F**) and OPA1, 225 MFN2, FIS1 or DRP1 (**Figures 4A–D**) in the mitochondrial fraction. LC3B-I (**Figure 3C**) also remained unchanged while LC3B-II increased on average by 30% over time, (main effect of time, P<0.05; **Figure 3D**). In a similar fashion, BNIP3 increased on average by 25% in the mitochondrial fraction (main effect of time, P<0.05 **Figure 3B**). The ratio between LC3B-II and LC3B-I remained unaltered in response to the intervention (**Figure 3E**).

## Discussion

We and others have previously reported that administration of T increases lean mass and aerobic capacity in human subjects (4, 26). However, the molecular mechanisms by which T elicits adaptations in skeletal muscle remains poorly understood. Here we report that T provision in young women did not modulate total levels of key proteins involved in androgen signaling/metabolism, mTORC1-signaling, ubiquitin and autophagy-mediated protein degradation and mitochondrial remodeling. Our findings thus suggest that increases in skeletal muscle size and mitochondrial function following T exposure are not related to altered expression of key factors regulating these processes.

In skeletal muscle, ARs are dispersed throughout the cytoplasm but predominantly located in resident muscle stem cells and myonuclei (34, 35). ARs are not only responsible for mediating the anabolic effects of T (10, 36), but are also critical for muscle fiber remodeling following chronic RT (37, 38). In males, administration of T is commonly accompanied by increased intramuscular AR content (13, 14, 17, 35, 39), but no study has previously examined whether such change occur in young women. In the present study, despite raising T serum concentrations ~ fivefold above basal levels, AR protein expression remained unchanged, a somewhat unexpected finding given that basal muscle AR content is lower in women than in men (40). The lack of change could however be related to the moderate dosage provided (10 mg daily), the route of administration (transdermal), or that AR levels were altered in a transient manner and had returned to a basal state at the post-biopsy timepoint. The latter is supported by work from Ferrando and colleagues showing elevated AR protein levels 4-weeks after the onset of T administration, but these had returned to pre-treatment levels 20 weeks later (17). Nonetheless, in accordance with our findings, AR protein levels were unaltered also in old women receiving oral oxandrolone (7.5 mg twice a day) for 14 consecutive days, whereas men in the same study displayed significant increases (39). Accordingly, rodent data suggest that ARs are dispensable for normal muscle development in females, but not in males (41). It is from our data therefore evident that an augmented intramuscular AR content is not critical in women for mediating the anabolic effects of elevated T levels. This finding may highlight a sexual dimorphism given that male subjects consistently display contrasting results. Furthermore, with regard to T metabolism, we also measured intramuscular content of 5α-reductase, the enzyme responsible for converting T into the highly potent metabolite dihydrotestosterone (DHT) (42). As both groups displayed slightly increased 5α-reductase protein content (main effect of time), we interpreted this as an effect not specific to T provision and that changes in muscle size cannot be explained by an altered capacity to metabolize T locally in the tissue. This aligns well with other studies questioning the anabolic effect of DHT in human subjects (43). On the other hand, we cannot completely rule out that other enzymes involved in androgen metabolism were affected by the current treatment, i.e., aromatase. Another factor that may have played a critical role for muscle adaptation here is the intramuscular concentration of T and other androgens, as discussed in a recent review (44). We did however not measure intramuscular levels of androgens in the present study but this is an important aspect that needs to be addressed by future work.

Studies in cultured cells and animal muscle have provided compelling evidence that T provision stimulates anabolism in an mTORC1-dependent manner (10–12), whereas data gathered from human trials have yielded inconsistent results (13, 14). To improve our understanding of how anabolic signaling may regulate muscle growth following T exposure, we measured the total expression of key proteins involved in the mTORC1-pathway. While most protein targets remained unchanged, only RPS6 displayed a significant pre-to-post increase in the T group, but this finding should be interpreted with caution as no interaction effect was present (main effect of time and group). Nevertheless, this may represent a potential mechanism by which T administration stimulates an increase in muscle mass. However, it is still possible that acute activation of this signaling cascade in response to anabolic stimuli, such as contractile activity and/or nutrients, was altered with T provision. In this regard, Gharahdaghi et al., 2019 demonstrated that T therapy potentiated acute resistance exercise-induced mTOR^Ser2448^ and RPS6^Ser235/236^ phosphorylation in older men (14). An enhanced response to each exercise session performed during the intervention may therefore explain how T provision stimulated muscle growth in our cohort. However, the observations of Gharahdaghi et al., 2019 were in a cohort of elderly men, in which a blunted signaling response due to potential anabolic resistance may have confounded the results. Future studies are therefore warranted to determine if a similar effect also exists in young individuals who are sensitive to anabolic stimuli. Similarly, whether T provision has a synergistic effect on the acute signaling response following nutrient intake is yet to be determined, but it has been reported that T provision does not further enhance the stimulatory role of amino acids on rates of muscle protein synthesis in human muscle (18), indicating that such effect would be of less importance to the present findings.

In humans, T provision is suggested to increase muscle mass in part by suppressing rates of muscle protein degradation (16–18), a process largely governed by muscle-specific E3 ligases MAFbx and MuRF-1 (20, 21). We therefore assessed the expression of these proteins together with the newly discovered E3 ligase UBR5. However, none of these targets were altered in response to T treatment. This would either indicate that transient changes in protein abundance were missed due to the current study design, or that muscle hypertrophy following T exposure is not related to changes in UPS-mediated protein breakdown (45). Regardless, our findings contrast recent work, in which reduced levels of MAFbx and MuRF-1 were found in hypogonadal men after they underwent 24-weeks of TRT (22). However, discrepancies between studies may be explained by differences in study population (young active women *vs* old hypogonadal men) and the dosage provided (10 mg *vs* 50-100 mg daily). Beyond this, conflicting findings may also be explained by endogenous androgen production. As such, it seems that rates of protein breakdown are suppressed by T provision only if endogenous concentrations are in the hypogonadal range and not when subjects are transitioning from the physiological to the supraphysiological range (9). Given that T levels were raised ~ fivefold above basal levels here, this notion seems to hold true also for women. It is however important to consider that the expression pattern of these E3-ligases might not fully reflect changes in proteasomal protein breakdown or muscle mass (46).

Another contributor to the overall protein balance in skeletal muscle is the lysosomal-autophagy pathway (47). Whether administration of T alters the autophagic process remains poorly understood and has, to our knowledge, not previously been investigated in human muscle. Current literature consists exclusively of animal studies where markers of autophagy have been assessed in response to surgical castration, which evokes rapid and profound changes in muscle mass, therefore providing little relevance to human subjects under free-living conditions. Nonetheless, castration-induced muscle atrophy is associated with increased phosphorylation of AMPK^Thr172^ and elevated LC3B-II content, as well as decreased phosphorylation of ULK1^Ser757^ and decreased p62 expression, which together indicate robust activation of the autophagy pathway (24, 25). The link between androgen levels and autophagy is further strengthened by the complete reversal of this effect once androgen levels are restored following castration (24, 25). In the present study, we did not find any alterations in total protein expression of AMPK, ULK1 or p62, which implies that the autophagy-lysosomal pathway did not mediate changes in muscle size following exogenous T provision. The present observation also opposes the notion that this pathway could be involved in the improved capacity to re-utilize amino acids in the fasted state, as previously observed here (17, 23). It must however be pointed out, once again, that the present study does not entail any information on the activation of this pathway in relation to acute stimuli, i.e., exercise, nor does it contain data on the transcriptional level. Thus, some aspects of this pathway with regards to muscle adaptation following T administration remains unanswered and requires additional study.

Mitochondrial remodeling through increased mitophagy, is crucial for maintaining a healthy mitochondrial network and impairments of this process are associated with declines in muscle size and function (48). During mitophagy, mitochondria are first separated from each other through fission, then tagged for removal by specific receptor proteins and later engulfed by autophagosomes for transport to the lysosome (48, 49). Interestingly, mice deprived of androgens displayed attenuated increases in oxidative capacity following chronic exercise, a phenomenon thought to be related to disrupted BNIP3-mediated mitophagy (50). While exercise training itself is sufficient to promote remodeling of the mitochondrial network (51, 52), preliminary evidence in mice suggest that exercise in combination with T provision promotes even greater activation of this system (29). Based on this, we reasoned that previously observed improvements in muscle oxidative capacity could be explained by a similar mechanism (26). We therefore assessed if markers of mitochondrial remodeling were influenced by T exposure. However, we did not observe any changes in markers associated with mitophagy or mitochondrial fission on the subcellular level. There were however some increases of BNIP3 and LC3B-II protein content (main effect of time), but these changes were not related to T provision *per se* and could potentially be related to subtle changes in subject´s training status, although we find this unlikely since exercise habits were well-maintained throughout the intervention (31). In addition, no effects were observed in MFN2 and OPA1 protein levels, two key regulators of mitochondrial fusion shown to be highly responsive to T exposure in rodent skeletal muscle (29). However, in this case, conflicting findings are likely due to profound differences in dosing regimens across species, in which serum T concentrations were raised 25-100 times above control level in the previously mentioned study (29).

Despite assessing several well-known regulators of muscle protein turnover, mitophagy and mitochondrial fission-fusion in the present study, it still remains to be determined by which molecular events T administration elicits hypertrophy and improved mitochondrial quality as we were unable to provide any clear mechanistic links here. One of the limitations of the present study is that we only reported chronic effects in muscle samples collected pre-post intervention. We therefore cannot exclude out that T administration, 1) transiently altered total protein abundance, 2) modulated the acute signaling response to exercise and/or nutrients, 3) influenced these regulating factors in a muscle fiber-type specific manner, 4) induced changes only at the transcriptional level. It is also possible that the sample size in the present study precluded us from detecting small but biologically relevant changes in total protein levels. To put our findings in light of the current literature is difficult as several inconsistences exist, which is reflective of the large variability among studies in terms of sex (men *vs* women), age (old *vs* young), endogenous androgens (hypo- *vs* eugonadal), route of administration (injections *vs* transdermal) and length of treatment. Nonetheless, the present study sheds important light on the effects of T provision on skeletal muscle in females and provides insights into their molecular underpinnings.

In summary, improvements in muscle size and oxidative capacity following 10-weeks of T administration in young women (4, 26, 31), cannot be explained by changes in protein expression related to muscle protein turnover or mitochondrial remodeling.

## Data Availability Statement

The original contributions presented in the study are included in the article/supplementary material. Further inquiries can be directed to the corresponding author.

## Ethics Statement

The studies involving human participants were reviewed and approved by Stockholm (2016/1485-32, amendment 2017/779-32). The patients/participants provided their written informed consent to participate in this study.

## Author Contributions

AL, BE, and WA were responsible for the conception and design of the study. OH, MM, and WA were responsible for data acquisition, data analysis and interpretation. OH drafted the manuscript. All authors provided intellectual content, contributed to the revision of the manuscript, and approved the final version to be published.

## Funding

This work was supported by the International Athletics Foundation, the Swedish Research Council (2017–02051), the Swedish Research Council for Sport Science (P2018-0197 and P2019-0098), the Karolinska Institute, and the Swedish Military Research Authority (Grant No. AF 922 0916). MM and WA were supported by Early Career Research Grants from the Swedish Research Council for Sport Science (D2017-0012 and D2019-0050, respectively).

## Conflict of Interest

The authors declare that the research was conducted in the absence of any commercial or financial relationships that could be construed as a potential conflict of interest.

## Publisher’s Note

All claims expressed in this article are solely those of the authors and do not necessarily represent those of their affiliated organizations, or those of the publisher, the editors and the reviewers. Any product that may be evaluated in this article, or claim that may be made by its manufacturer, is not guaranteed or endorsed by the publisher.
